# Negative-control-anchored urinary microbiome profiling with absolute 16S quantification: a pilot study in newly diagnosed, treatment-naive bladder cancer and healthy individuals

**DOI:** 10.1093/femsle/fnag020

**Published:** 2026-02-17

**Authors:** Tomaž Accetto, Katja Strašek Smrdel, Milena Taskovska, Marjanca Starčič Erjavec, Tomaž Smrkolj, Katja Seme, Mateja Erdani Kreft

**Affiliations:** Department of Microbiology, Biotechnical Faculty, University of Ljubljana, SI-1000 Ljubljana, Slovenia; Institute of Microbiology and Immunology, Faculty of Medicine, University of Ljubljana, SI-1000 Ljubljana, Slovenia; Clinical department of Urology, University Medical Centre Ljubljana, SI-1000 Ljubljana, Slovenia; Chair of Surgery, Faculty of Medicine, University of Ljubljana, SI-1000 Ljubljana, Slovenia; Department of Microbiology, Biotechnical Faculty, University of Ljubljana, SI-1000 Ljubljana, Slovenia; Clinical department of Urology, University Medical Centre Ljubljana, SI-1000 Ljubljana, Slovenia; Chair of Surgery, Faculty of Medicine, University of Ljubljana, SI-1000 Ljubljana, Slovenia; Institute of Microbiology and Immunology, Faculty of Medicine, University of Ljubljana, SI-1000 Ljubljana, Slovenia; Institute of Cell Biology, Faculty of Medicine, University of Ljubljana, SI-1000 Ljubljana, Slovenia

**Keywords:** bladder cancer, midstream urine, microbiota, urinary tract infection, next-generation sequencing, nonmetric multidimensional scaling, absolute quantification, negative controls, low biomass

## Abstract

Recent studies utilizing 16S rRNA amplicon sequencing have challenged the notion of urine sterility, yet urine is a low-biomass specimen in which apparent community profiles can be strongly influenced by background signal from reagents and processing. To address this interpretability gap, we integrate culture-independent absolute 16S rRNA gene quantification with urinary 16S amplicon sequencing in a negative-control-anchored workflow. Bacterial load provides a biomass-aware quality control gate that defines interpretable low-biomass thresholds and objective exclusion criteria. As a pilot application, we compared midstream urine collected prior to instrumentation from healthy volunteers and newly diagnosed bladder cancer (BC) patients, quality filtering retained 29 controls and 5 BC cases. Samples > 10^6^ copies/ml typically produced > 10 000 reads; near 10^5^ copies/ml read counts dropped sharply yet remained distinguishable from background. Thirteen negative controls (V3–V4 PCR and stabilization buffer; median 90, mean 124 reads) supported excluding samples with < 1000 reads. Median bacterial load was lower in BC than in controls (7.0  × 10^3^ vs 1.07 × 10^6^ copies/ml), although not significant in this underpowered cohort (*P *= 0.07). This cohort-size-independent framework enables load-based triage for sequencing, reduces background-driven over-interpretation in low-biomass urine datasets, and supports modeling bacterial load as a covariate or stratifier in future studies of the bladder cancer microbiome.

## Teaser

We developed a negative-control anchored urine microbiome workflow, using absolute bacterial load to improve low-biomass data reliability and explore differences between healthy individuals and bladder cancer patients.

## Abbreviations

BC: Bladder Cancer
UTI: Urinary Tract Infection
rRNA: Ribosomal RNA
DNA: Deoxyribonucleic Acid
OTU: Operational Taxonomic Unit
PCR: Polymerase Chain Reaction
NGS: Next-Generation Sequencing
NMDS: Nonmetric Multidimensional Scaling

## Introduction

Traditionally, the urine has been considered sterile, and the fast-growing bacteria like *Escherichia coli* were only cultivated from it in connection with urinary tract infections (UTIs) (Whiteside et al. 2015). This was challenged with culture-independent 16S rRNA amplicon sequencing in the past decade which readily detected bacterial communities (Whiteside et al. 2015). This gave incentive to develop more sophisticated culturing procedures (the enhanced quantitative urine culture) (Hilt et al. 2020), which largely confirmed the existence of permanent bacterial microbiota in urine (Price et al. 2020). The urine communities of healthy women are best characterized and are far from uniform but are at the same time less species-rich and diverse than the gut microbiota (Price et al. 2020). They have been divided into urotypes, based on the more than 50% abundance of an individual taxon (genus). The most frequent are *Lactobacillus, Streptococcus, Gardnerella*, and *Escherichia*, while there is a considerable fraction of samples where other bacteria are dominant or none of them reaches the 50% (Price et al. 2020). Though the connection of the urotypes to lifestyles, diets, and fluid intake, as well as microbial community diurnal dynamics are unknown, the evidence linking age to different urotypes is beginning to emerge (Price et al. 2020). The urinary microbiota of men is less well known, but the main urotypes seem to be similar though the most prevalent one may be *Corynebacterium* instead of *Lactobacillus* (Perez-Carrasco et al. 2021). The average density of the urinary microbiota by culturing was approximated at less than 10^4^ colony-forming units/ml urine with the median of 85 CFU/ml (Pearce et al. 2014) and may be easily contaminated from other sources like the gut or the vagina during sampling. Thus, considerable effort was devoted to clarify which type of urine samples are most adequate, the consensus being that the urine from catheterized urine is preferable as the contamination from urethra, skin, and genital tract is reduced relative to midstream urine samples and the procedure is not as invasive as in suprapubic aspiration that would potentially result in even less contaminated samples (Perez-Carrasco et al. 2021). However, use of catheterized urine may result in significant (Bajic et al. 2020) or only subtle (Oresta et al. 2021) differences in community structure relative to those obtained from voided or midstream urine samples, respectively. The role of the urinary microbiota is not exactly known. It was suggested that its function may be, like in the gut, in pathogen exclusion and immune system development (Whiteside et al. 2015). Yet, because nutrients are scarce in the bladder and come mainly from the glycosaminoglycans secreted by urothelium (Lacerda Mariano and Ingersoll 2020), leading to sparse microbiota, these functions may not be on par to those in the gut.

Bladder cancer (BC) is the second most common genitourinary tract cancer. Over the past years, it has become an important healthcare problem because of its malignant potential, the costs of treatment and follow-up (Sung et al. 2021). It is known that schistosomiasis and chronic UTI are risk factors for BC (Gouda et al. 2007). The role of the urinary microbiota in its establishment and progress has been approached by several studies. Some of them centered on the cancerous tissue itself that was removed during transurethral resection and identified *Akkermansia, Bacteroides, Klebsiella, Clostridium*, and *Enterobacter* as overrepresented there (Mansour et al. 2020), but other researchers identified a plethora of other nonoverlapping genera (Liu et al. 2019). Other studies focused on the urine (Popović et al. 2018, Oresta et al. 2021) and noted that its microbiota had high variability among individuals and that the healthy controls were indistinguishable from BC patients either on ordination plots, multivariate statistical tests, and most of the sample diversity indices. They nevertheless found taxons that exhibited differential abundances in cancerous relative to control samples, e.g. *Veillonella* and *Corynebacterium*, which were, however, enriched in one study but depleted in the other (Popović et al. 2018, Oresta et al. 2021). The patients in these studies were mixed; some were recurring cases and some had the first occurrence of the BC. All of the above studies yielded insight into the relative abundance of the most prominent genera of the healthy and BC linked urinary microbiota, but at the same time, most did not address the bacterial load of the samples. Absolute bacterial-load information is essential to (i) determine whether a profile is interpretable above negative controls and (ii) distinguish true microbial depletion from purely compositional shifts.

In this study, we therefore provide an analysis workflow anchored to negative controls that integrates absolute 16S rRNA gene quantification into 16S amplicon sequencing of midstream urine. Specifically, we (i) establish an empirical bacterial-load range that routinely yields robust sequencing output in urine; (ii) define objective exclusion criteria grounded in negative controls; and (iii) apply this framework as a treatment-naive, pre-instrumentation pilot comparison between newly diagnosed BC patients and healthy controls.

## Patients and methods

### Participant recruitment, their characteristics, and sample collection.

Written consent was obtained from participants prior to recruitment. The study was approved by the National Ethics Committee of the Republic of Slovenia, No. 0120-27/2019/8.

In a prospective pilot study, we included 6 patients (male and female), 18 years old or more, with a diagnosis of BC (Table 1) that were treated at the Clinical department of Urology, University Medical Centre Ljubljana, Slovenia. Patients with a past history of BC, UTI, urolithiasis, enlarged prostate, with urinary catheter, radiotherapy of pelvis, previous urologic, abdominal, and gynecological surgery, instrumentation of urinary tract (cystoscopy, ureterorenoscopy, urinary tract drainage, and catheters) were excluded from the study.

**Table 1:** Characteristics of patients with BC.

Patient ID.	Gender	Age	Main complaint	Comorbidities	Urine culture	Antibiotic	Cystoscopy	Cytology	Treatment 1	Histopathology	Treatment 2	Histopathology 2	Treatment 3
122	M	75	Frequent urination	0	Neg	NA	Bladder tumor	Positive	TURBT	pT2 HG urothelium carcinoma	Rejects treatment	NA	
1322	M	60	Hematuria	0	Neg	NA	Bladder tumor	Positive	TURBT	pT2 HG urothelium carcinoma	Trimodal treatment	NA	
1023	M	76	Bladder tumor	Diabetes	*E .faecalis*	Nitrofurantoin	Bladder tumor	Positive	TURBT	pTa HG urothelium carcinoma	re-TURBT	Chronic inflammation after TURBT	BCG x6
1123	M	87	Bladder tumor	BPH	Neg	NA	Bladder tumor	Negative	TURBT	pTa HG urothelium carcinoma	re-TURBT	Chronic inflammation after TURBT	BCG x6
1223	F	59	Bladder tumor	0	Neg	NA	Bladder tumor	Positive	TURBT	pT2 HG urothelium carcinoma	Radical cystectomy + lymphadenectomy	pT3 HG ugothelium carcinoma, positive lymph nodes	Adjuvant chemotherapy
2123	F	66	Bladder tumor	Biabetes	Neg	NA	Bladder tumor	Negative	TURBT	CIS	re-TURBT	*	

Abbreviations: BCG, Bacillus Calmette–Guérin intravesical; BPH, benign prostatic hyperplasia; CIS, carcinoma in situ; F, female; M, male; NA, not applicable; TURBT, transurethral resection of bladder tumor.

In the control group, we included 22 female and 12 male volunteers (Table S1), 18 years old or more, that were recruited at the Clinical department of Urology, University Medical Centre Ljubljana, Slovenia. The participants in the control group were individuals without active or past urinary tract pathology, without history of urinary tract instrumentation or surgical procedures in the pelvis.

Urine samples were collected into sterile urine containers and transported to the Institute of microbiology and immunology, Faculty of Medicine, University of Ljubljana, Slovenia, using standard protocol. Each participant provided three urine samples for study purposes, which were derived from initial and mid-stream urine, each with 15–50 ml of volume. We asked all participants to follow the standardized rules for urine collection. Patients cleaned their external urethral meatus with three sterile gauze tampons and collected an initial urine sample in the first container and the mid-stream in the second and third containers. The first and second containers (50-ml conical tubes) contained 2.1 ml of urine conditioning buffer (Zymo Research), which preserves DNA and RNA in urine at room temperature. The sample from the first container—initial stream was used as a control for the detection of possible contamination of the external urethral meatus/genitalia. The mid-stream samples were considered as the most representative samples of urine, without contaminants from external urethral meatus or genitalia, and data arising from these samples are presented in the Result section. The third container was sent for urine culture as well as for clinical purposes: diagnosis and treatment of UTI. In all patients, specimens were collected before instrumentation (e.g. cystoscopy).

During ambulatory evaluation, we collected data about age, gender of participants, leading cause for urology consultation, co-morbidities, medications, history of surgical procedures, diagnostics (CT urography, ultrasonography, cytology, and cystoscopy), and treatment.

### DNA isolation

DNA was extracted using the ZymoBIOMICS DNA Miniprep kit (Zymo Research). Urine samples were centrifuged at 3000 g for 15 minutes. The supernatant was removed without disturbing the pellet. ZymoBIOMICS Lysis Solution was added to the pellet to a final volume of 800 µl and transferred to a ZR BashingBead Lysis Tube. Tubes contained 0.1 and 0.5 mm beads for optimal lysis of Gram-negative and Gram-positive bacteria. For positive control of extraction, the ZymoBIOMICS Microbial Community Standard D6300 (Zymo Research) was used. As a negative control of the extraction urine conditioning buffer was included in each extraction session performed. Homogenization was performed on the MagNA Lyser instrument (Roche) with seven homogenization cycles at 5000 Hz for 45 seconds. Between cycles, samples were cooled at 4°C for 5 minutes. Further processing was performed according to the manufacturer’s instructions. Positive and negative controls of extraction were later used in real-time PCR and amplicon sequencing along with the rest of patients’ samples. After extraction, the amount of extracted double-stranded DNA was determined using the Qubit dsDNA (High sensitivity) Assay Kit (Invitrogen).

### Bacterial load of the urine samples

Bacterial density in the samples was assessed using absolute 16S gene counts. Absolute 16S rRNA gene quantification was used as an a priori quality indicator for low-biomass urine sequencing and to interpret sequencing output relative to negative controls. A cloned *Escherichia coli* 16S rRNA standard (Tib MolBiol) was used to generate a calibration curve with dilutions of 10^7^ to 10 copies/reaction. Quantitative real-time PCR using 16S Mastermix Complete (Molzym) was performed to estimate the 16S gene counts. Each real-time PCR run included a standard dilution series for quantification and both positive (bacterial DNA) and negative controls (molecular biology grade water) to ensure assay performance and exclude contamination, in addition to samples and extraction controls.

### 16S rRNA gene V3–V4 amplicon sequencing

16S rRNA sequencing was performed according to the 16S Metagenomic Sequencing Library Preparation protocol for the Illumina MiSeq System (Illumina). A minimum of 5% PhiX was used as an internal control for each sequencing run. As recommended, MiSeq Reagent Kit v3 (600 cycles) cartridge (Illumina) was used for one sequencing run. Samples were sequenced with paired-end reads of 2 × 300 bp. Negative and positive extraction controls and a negative control of 16S RNA amplification process were subjected to the sequencing workflow to assess potential cross-contamination during sample processing.

### Bioinformatic and statistical analysis

The Illumina reads were processed using the UPARSE pipeline (Edgar 2013) and the taxonomies of operational taxonomy units (OTUs) were inferred with the RDP v16 training set that contains 16S rRNA gene sequences of the type strains of bacterial species. Contamination from human sequences was removed using Bowtie2 (Langmead and Salzberg 2012) and *Homo sapiens* genome assembly GRCh38-based index. When merging sequence reads, the minimum length allowed was 380 bp. The OTUs sequence alignments were inspected for any remaining anomalous sequences and the OTU phylogeny reconstruction was done using PHyML (Guindon et al. 2010). The sample metadata, taxonomy table and operational taxonomy unit tables were then imported and analyzed using phyloseq R package (McMurdie and Holmes 2013) where α-diversity in relation to BC was assessed using the Shannon index. Diversity among healthy and BC groups was analyzed using nonmetric multidimensional scaling and the distances computed with UniFrac or Bray–Curtis indices. R, version 4.0.3 was also used for statistical analysis.

## Results

Based on quantitative real-time PCR targeting the 16S rRNA gene, the estimated bacterial load was sufficient for subsequent molecular analyses. The bacterial load in the extraction negative control was predominantly below 10² bacterial cells per milliliter of sample. Nevertheless, all samples, including the extraction controls, were subjected to 16S rRNA gene amplicon sequencing.

### The sensitivity and validity of 16S amplicon sequencing

**Figure 1:**
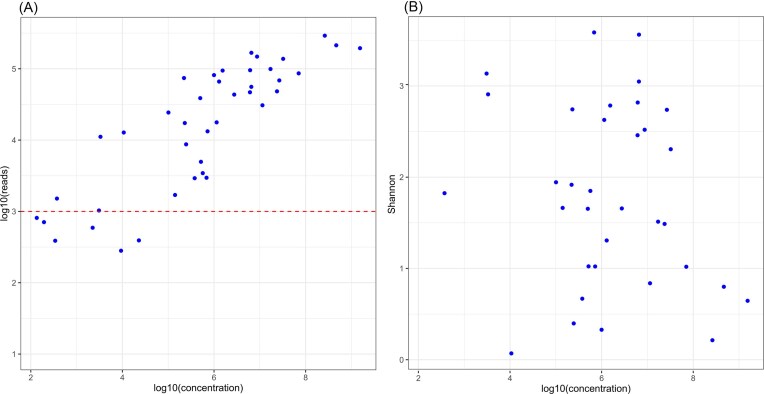
Obtained 16S rRNA amplicon reads (A) and Shannon diversity index (B) per sample after quality filtering relative to 16S rRNA gene concentration in urine of sample as determined by real-time PCR.

Since the urinary microbiota is sparse, we tested the ability of the amplicon sequencing protocol used to reliably deliver a meaningful amount of data. In our setting, the concentration of 16S rRNA gene of more than 10^6^/ml of urine yielded more than 10 000 sequences (Fig. 1A). Thus, given that median of 16S rRNA gene copies per cell is 6 in bacteria (Stoddard et al. 2015), the protocol routinely gave rich results at approximately > 1.7 × 10^5^ bacterial cells per ml of urine. Around half of the samples were, however, below this threshold. Since thirteen negative controls of V3-V4 16S PCR and urine stabilization buffers had median and average counts of 90 and 124 reads, respectively, we decided to exclude samples producing less than 1000 reads from analysis. This resulted in the exclusion of one cancer patient and five healthy controls, keeping samples of 34 individuals (5 BC patients and 29 healthy volunteers) for further analysis. The Shannon diversity index was widely variable across measured concentrations of 16S rRNA gene (Fig. 1B) which suggests there were no appreciable influences from DNA and species rich samples to other samples during processing. Thus, we were able to characterize both species-rich and -poor communities across a large interval of bacterial concentrations. The samples containing mock communities yielded bacterial compositions close to the manufacturer’s specifications and no deficiency in DNA isolation from Gram-stain positive bacteria was evident. In six cases, the urine cultures were positive and isolates identified. In five out of six cases, the isolated genera were observed also using the 16S rRNA NGS approach.

### The healthy control urinary microbiota

Using the > 50% reads measure to delineate urotypes, the 22 healthy women were of the *Lactobacillus* (13), *Gardnerella* (2), mixed (3) and of *Bifidobacterium* (1) urotype, while for three, sequencing did not yield results. Other abundant genera were *Prevotella, Finegoldia, Streptococcus, Escherichia*, and *Peptoniphilus*. These observations were in line with earlier results using catheterized urine samples (Price et al. 2020). The men’s urotypes were mixed (9), *Gardnerella* (1), and two samples with no sequencing results. The detailed shares of genera can be seen in Table S1. The alpha diversity of samples as judged by the Shannon metric varied wildly from 0.21 where *Lactobacillus* dominated with 96% of reads to 3.59, with the most abundant genus *Prevotella* encompassing only 8.8% of all reads and additional 22 genera in 39 OTUs (each above 0.5% of reads) being the rest. Generally, in men, the urine microbiota tended toward higher diversity.

### Urinary microbiota of the BC patients

**Figure 2:**
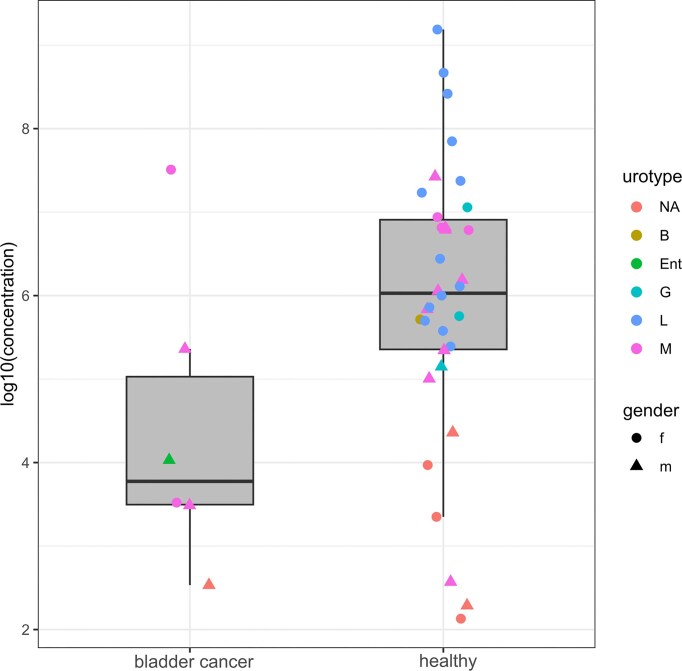
Bacterial load of the urine samples of bladder cancer patients without prior genitourinary tract conditions and medical interventions and healthy control group without prior genitourinary tract conditions and medical interventions. The concentration is given in 16S rRNA gene copies per ml of urine. The letters correspond to the urotype of the samples: L, *Lactobacillus*; M, mixed; G. *Gardnerella*; Ent, *Enterococcus*; B, *Bifidobacterium*, NA, not available-the samples yielded no sequencing result.

**Figure 3:**
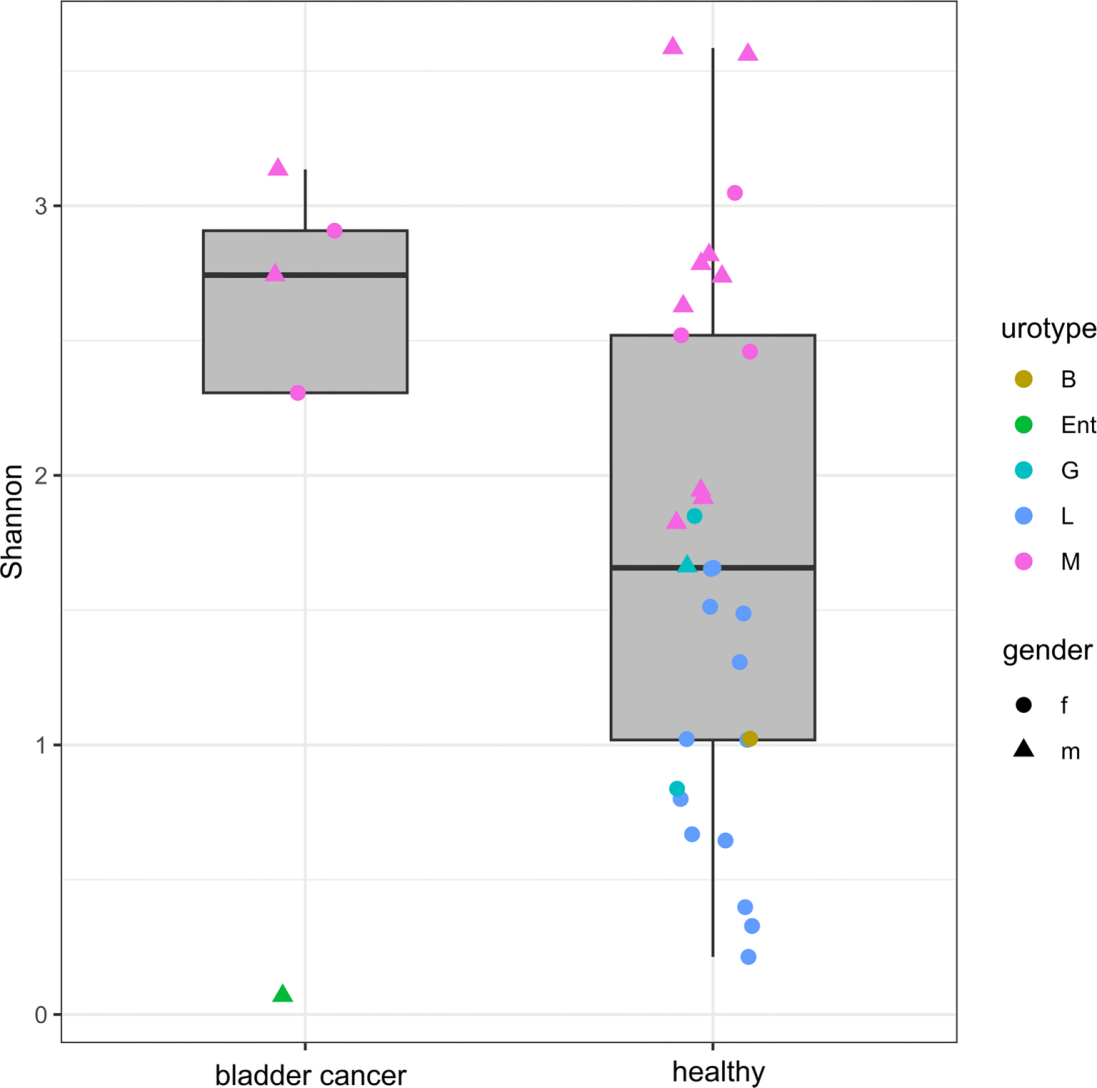
Intra sample diversity as given by Shannon metric in bladder cancer patients and healthy control group. The letters correspond to the urotype of the samples: L, *Lactobacillus*; M, mixed; G. *Gardnerella*; Ent, *Enterococcus*; B, *Bifidobacterium*.

**Figure 4:**
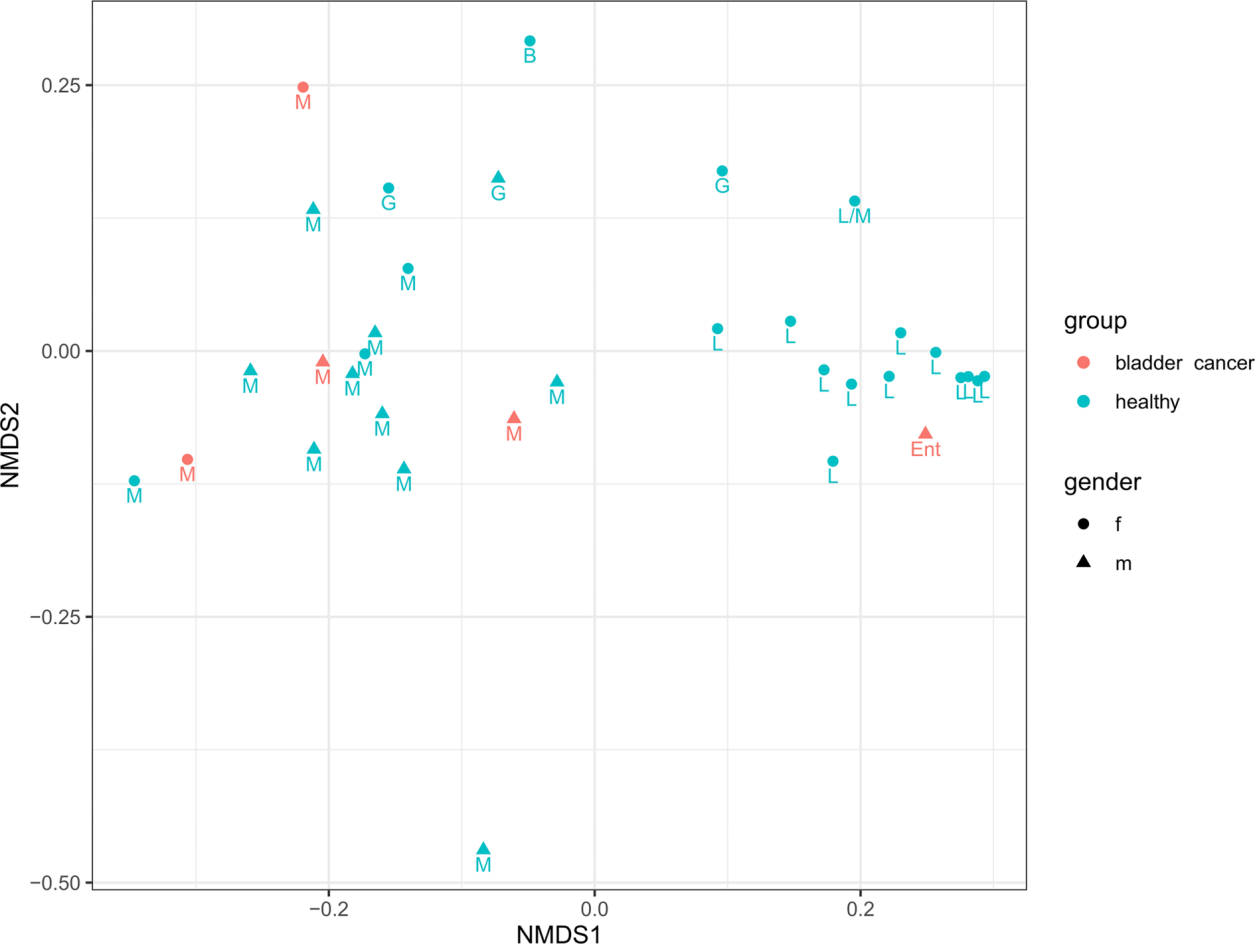
A nonmetric multidimensional scaling plot using the weighted UniFrac differences among samples. The gender is given by the shape and the group identity by the color of the symbols. The letters correspond to the urotype of the samples: L, *Lactobacillus*; M, mixed; G. *Gardnerella*; Ent, *Enterococcus*; B, *Bifidobacterium*.

The BC patients were all presenting with cancer for the first time, so although the samples were not many, they represented the urinary microbiota that had not been skewed by earlier interventions (e.g. cystoscopy). The first attribute of this cancer urine microbiota seemed its low abundance (Fig. 2) as the median of 16S rRNA gene copies per ml of urine in the BC group was 7010, while it was 1.07 × 10^6^ in the control. Also, one sample from BC patients yielded no amplification products in NGS due to low bacterial concentrations. The 16S rRNA gene concentrations of the BC patients and healthy volunteers were not normally distributed as judged by the Shapiro test, so the Mann–Whitney U test was used to assay for possible difference in 16S rRNA concentrations. The test rejected that BC samples contained statistically significantly lower concentrations of bacteria (*P *= 0.07). Although underpowered for definitive inference, the observed magnitude of difference highlights why absolute quantification is necessary when comparing low-biomass urine profiles across groups. When examining the alpha diversity (Fig. 3), four of the BC patient samples were of the mixed urotype, rich with diverse bacterial genera (Table S1), while 99% of the reads obtained from the fifth were assigned to *Enterococcus*, a common urinary pathogen (Lacerda Mariano and Ingersoll 2020). Notably, both women with BC were of mixed urotype which was otherwise rare in healthy women (3 out of 19). When the ordination plot using NMDS and weighted UniFrac distance was made, the *Enterococcus*-containing BC sample was predictably split from other BC samples (Fig. 4). These other BC samples were interspersed among the healthy controls of the mixed urotype of men and women implying similar communities. The urinary microbiota of healthy women exhibiting *Lactobacillus* urotype formed a distinct grouping at the right, where the most *Lactobacillus* dominated samples were placed at the far right whereas samples containing significant admixtures of *Gardnerella, Bifidobacterium, Escherichia*, and *Streptococcus* (Table S1) were placed at the left side of this cluster. The *Enterococcus* dominated BC sample was near the *Lactobacillus* cluster because of the phylogenetic proximity of these two genera which both belong to the *Lactobacillales*. Quite similar clustering was also seen when the dissimilarity measure used for ordination was Bray–Curtiss (Fig. S1).

## Discussion

The principal contribution of this work is methodological: we provide an analysis framework anchored to multiple negative controls that integrates absolute 16S rRNA gene quantification into urinary 16S amplicon sequencing to address the interpretability problem inherent to low-biomass urine. By explicitly linking bacterial load to sequencing output and grounding exclusion thresholds in negative controls, this approach improves reproducibility and reduces background-driven over-interpretation in urinary microbiome studies.

Previous studies (Popović et al. 2018, Oresta et al. 2021) of the BC urine microbiota seemingly contradicted each other in the identification of enriched/depleted bacterial species. Yet, combining their evidence with ours it seems likely that at least two community states may exist: (i) a rich microbiota state resembling the mixed urotype communities of the healthy control group and (ii) a microbiota largely dominated by a single genus. The latter was already observed (Popović et al. 2018) but was excluded from the analysis because of inconsistency with other samples. It remains to be seen if these states are parts of the same urinary microbiota trajectory or stem from the fundamentally different starting points, but only studies with many more samples surveying patients through prolonged periods could resolve this. While the first state is frequently observed and is reminiscent of healthy male and female microbiota which is not separated from it in ordination plots, the second state poses questions of its provenance and importance in cancer progression. Is this a secondary infection caused by disruption originating from cancer or was the prolonged inflammation the cause of cancer? Our results suggest that the whole urine microbiota may often be depleted in bladder cancer patients as evidenced by 16S rRNA gene real-time PCR, which seemed to favor the first scenario. Yet, when considering the whole BC group as a homogeneous entity, urine microbiota depletion was not statistically significant. Importantly, the negative-control-anchored thresholding framework is cohort-size independent and can be directly applied to larger BC cohorts, where absolute bacterial load can be incorporated as a covariate or stratification factor to disentangle true microbial depletion from compositional effects. This finding, however, should be corroborated by further simple association of involving many more BC patients as there is a common belief of urologists that the presence of BC promotes growth and sequestration of bacteria on irregular tumor surface. In any case, the existence of more than one urinary microbiota state in BC patients complicates the simple association of urinary microbiota with BC and this should be done “carefully and with healthy skepticism” as suggested recently (Werneburg and Southgate 2024). Leaving aside the above discussion on community composition, we also observed substantial variation in the bacterial load of urine samples from both healthy individuals and BC patients. This variability may arise from a multitude of factors and warrants further investigation. Recognizing such heterogeneity may assist urologists in identifying microbiota-associated subtypes of bladder cancer, thereby informing patient stratification, prognostic assessment, and potentially contributing to the development of microbiota-targeted therapeutic strategies.

A limitation of this study is the small number of BC cases, which restricts statistical power for drawing disease-association conclusions; therefore, BC-related observations should be considered hypothesis-generating. Importantly, we developed a novel negative-control-anchored thresholding framework, which is independent of cohort size and can be readily applied to larger BC cohorts, where absolute bacterial load may be incorporated as a covariate or stratification factor to disentangle true microbial depletion from compositional effects.

## Supplementary Material

fnag020_Supplemental_Files

## Data Availability

The datasets generated and/or analyzed during the current study are publicly available available in the Sequence Read Archive (SRA) under the BioProject accession number PRJNA1398786.
