# Supplementary material for: Negative-control-anchored urinary microbiome profiling with absolute 16S quantification: a pilot study in newly diagnosed, treatment-naive bladder cancer and healthy individuals
Source: FEMS Microbiol Lett. 2026 Feb 17;373:fnag020. doi: 10.1093/femsle/fnag020 (PMC13017691; doi:10.1093/femsle/fnag020)
Supplement: fnag020_Supplemental_Files [file fnag020_supplemental_files.zip › Supplementary data captions.docx]

**Supplementary data captions**

**Table S1**. Patient and molecular data obtained in this study.

**Figure S1**. A Non-metric multidimensional scaling plot using the Bray-Curtiss differences among samples. The gender is given by the shape and the group identity by the color of the symbols. The letters correspond to the urotype of the samples: L, *Lactobacillus*; M, mixed; G. *Gardnerella*; Ent, *Enterococcus*; B, *Bifidobacterium*.
