# Supplementary figures and images for: Negative-control-anchored urinary microbiome profiling with absolute 16S quantification: a pilot study in newly diagnosed, treatment-naive bladder cancer and healthy individuals
Source: FEMS Microbiol Lett. 2026 Feb 17;373:fnag020. doi: 10.1093/femsle/fnag020 (PMC13017691; doi:10.1093/femsle/fnag020)

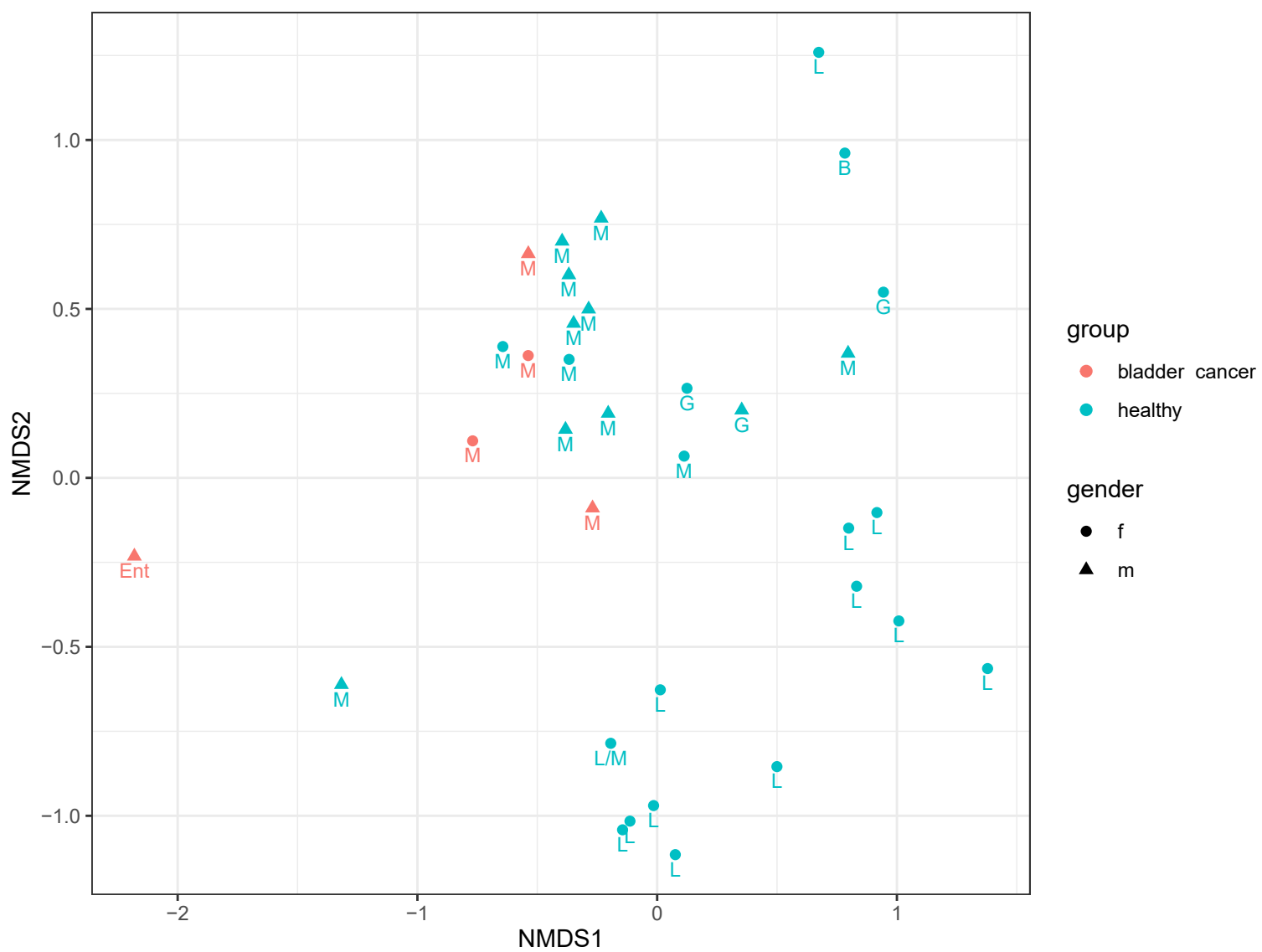

Supplement: fnag020_Supplemental_Files [file fnag020_supplemental_files.zip › figure_s1.pdf]
